# Evaluation of an activated carbon disposal system for safe disposal of model prescription sedative medications

**DOI:** 10.1038/s41598-020-59907-2

**Published:** 2020-02-19

**Authors:** Behnam Dasht Bozorg, William Fowler, Andrew Korey, Carter Anderson, Ajay K. Banga

**Affiliations:** 1 Center for Drug Delivery Research, Department of Pharmaceutical Sciences, College of Pharmacy, Mercer University, Atlanta, GA 30341 USA; 2 Verde Technologies, 12900 Whitewater Drive, Minnetonka, MN 55343 USA

**Keywords:** Environmental impact, Public health

## Abstract

Lack of a safe and convenient disposal method for expired and unused medications may lead to many problems such as accidental exposure, intentional misuse, and food and water contamination. Activated carbon can offer safe disposal of medications due to its highly porous structure, which exerts strong physical adsorption forces with chemicals. This study aimed to evaluate the efficiency of an activated carbon-based drug disposal system for deactivating three model sedative prescription medications. Deactivation was performed by mixing the medication, activated carbon, and tap water. Desorption was evaluated by exposing the deactivation system to wash-out solutions. Rapid, precise, accurate, and sensitive HPLC-UV method for each drug was successfully developed, validated and employed. Results of the 28-day deactivation study showed that on average, more than 94.00% of drugs were rapidly deactivated within 8 hours. All drugs reached more than 99.00% deactivation by the end of 28-day period. Desorption study demonstrated that all medications were retained by the system, with insignificant amount of drug (0.25%) leached into the washout solutions within 24 hours. In conclusion, activated carbon rapidly and successfully deactivated the medications tested, suggesting activated carbon-based drug disposal system provides a convenient, secure, and effective method for unused medication.

## Introduction

Prescription drug misuse and abuse, a major public health concern in the United States, is the intentional or accidental consumption of medicines without a prescription, for a purpose other than prescribed or to experience the feeling they may cause. In 2016, National Survey on Drug Use and Health (NSDUH) reported that 10.6 percent of people have used illegal substances in the past month, and 7.5 percent were reported to experience a drug use disorder in the past year^1^. Misuse of prescription psychotherapeutic drugs is relatively common in the United States and is second to marijuana as the most commonly used illicit drug^2^. In 2016, an estimated 6.2 million Americans aged 12 and over were reported to misuse psychoactive medications at least once in the past month, representing 2.3 percent of the population aged 12 and over^3^. Four main categories of prescription psychotherapeutic drugs include pain relievers, stimulants, tranquillizers, and sedatives^2^.

In 2016, approximately 294,000 people were reported to have misused prescription sedatives for the first time during the past year. This estimated number averages to about 800 initiates per day for prescription sedative misuse and 24.8 years was reported as the average age at first misuse occurrence among recent initiates^1^.

Prescription sedative medications are psychotherapeutics which are often prescribed to treat disorders such as insomnia. There are different subtypes to prescription sedatives including benzodiazepine sedatives (e.g., alprazolam, temazepam) and zolpidem products (e.g., Ambien^®^). The most common reported reason for the misuse of prescription sedatives among adults was to help with sleep (73.2%), which is still considered misuse if taken without a prescription, taken more frequently, or at higher doses than prescribed. Other reported reasons for misuse were to relax or relieve stress (12.0%), to feel better or get high (5.1%), to help with feelings or mood (3.9%), to experience the feeling the drug might cause (3.0%), and to enhance or reduce the effects of other drugs (1.3%)^4^.

Many patients report that they store unused or expired medications including prescription sedatives in their households. Recently, there has been more focus on the accumulation of unused medications in US households and its negative consequences on health outcomes, and safety of patients and the environment^5,6^. The main reasons that can cause patients to not use all the medicines dispensed include side effects, changes in dosage, medicine discontinuation, or expiration of medications. It has been reported that two-third of dispensed medications are not used, with a national projected cost range of $2.4B to $5.4B^5^. Unused prescription medications often accumulate in households and can be intentionally abused, inappropriately misused to self-medicate or accidentally ingested^7,8^.

Utilizing suitable techniques to discard expired, and unused medications in households will help to reduce risks associated with accidental exposure or intentional abuse and misuse. For safe and secure disposal, FDA suggests that consumers transfer their unneeded medicines to drug take-back events held by The U.S. Drug Enforcement Administration (DEA) or local law enforcement agencies. Although there is interest and participation in drug take-back programs, there is a lack of awareness and accessibility of on-going programs^9^. As an alternative, FDA recommends the following steps: Unused medicines should be mixed with an inedible substance such as used coffee ground, dirt, or cat litter; the mixture is then placed in a sealed plastic bag, and finally it is discarded in household trash. In case of potentially dangerous medications, they should be flushed down the toilet or sink once they are no longer needed^10^. However, other than the risk of these medications being misused or abused, improper disposal can result in contamination of food and water supplies. Recently, the presence of numerous pharmaceuticals and their metabolites in American waterways, groundwater, and even drinking water has been recognized as potentially dangerous^11^. Since water treatment systems currently are not capable to completely remove many pharmaceuticals from drinking water, it results in long-term exposure to traces of several medications which can be harmful. Moreover, drug take-back programs employ incineration, which can lead to toxic air emissions^12^. The inappropriate methods to discard unused and expired medications is a source of water and environment contamination which can be prevented^8^. Hence, a safe and efficient drug disposal system can be advantageous for household and healthcare use.

**Figure 1:**
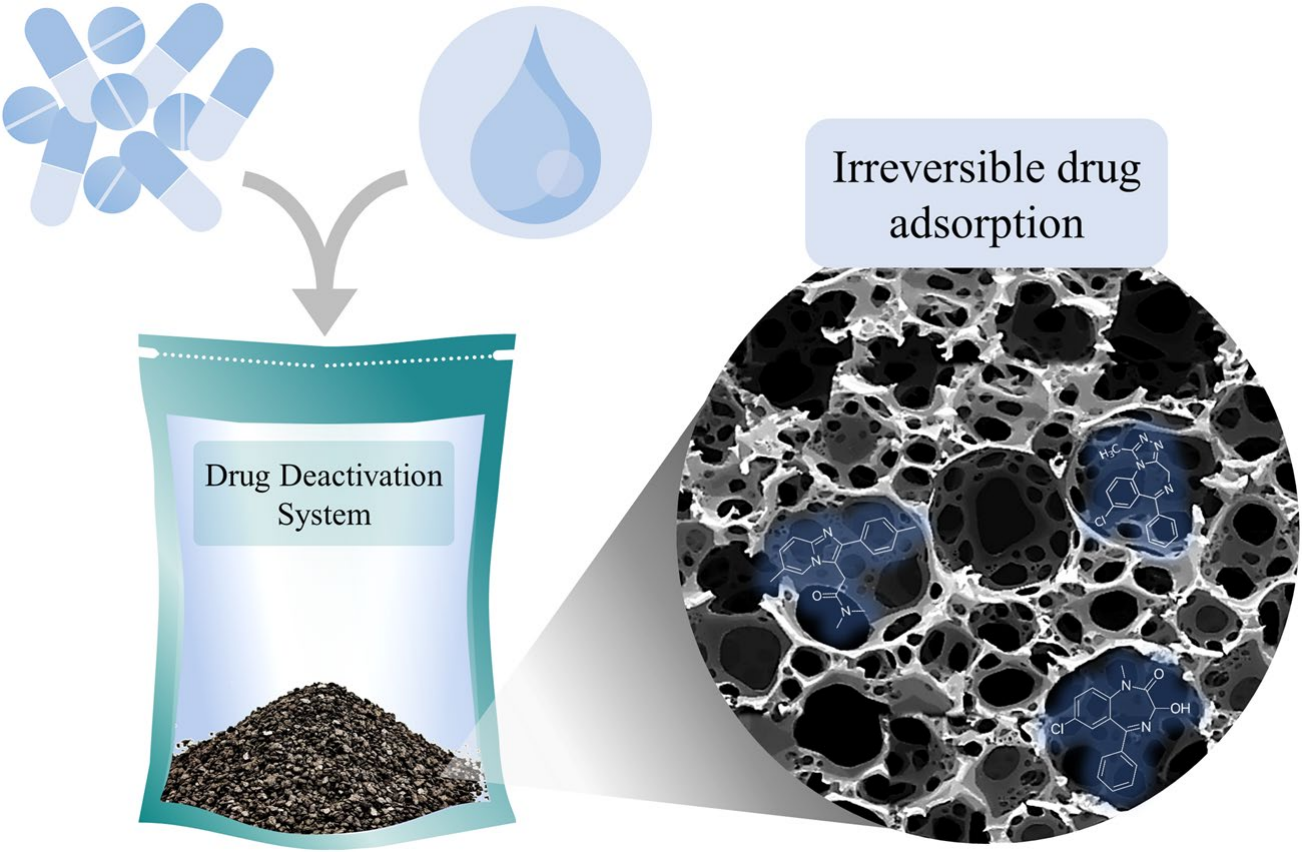
Schematic demonstrarion of the the investigated drug deactivation system.

Activated carbon is considered to be a universal adsorbent. It can inactivate chemical substances by adherence of an extremely thin layer of the compounds to the large surface area (500 to 1500 g/m^2^) of the carbon due to its highly porous structure which exerts sufficient adsorption and retention of pharmaceutical compounds^13,14^. It is recommended for treating drug overdose or chemical poisonings in emergency situations due to its strong adsorption properties^15^. Activated carbon has been successfully used in drug deactivation^16,17^. For this study, the term “deactivation” is used to indicate the irreversible physical adsorption of the drug to the activated carbon. The deactivation system tested in this study, is fashioned from a pre-packed re-sealable outer plastic pouch containing granulated activated carbon utilizing molecular adsorption technology (MAT12^®^), enclosed in a water-soluble film. In this design, medications are to be placed in the pouch, followed by the addition of tap water, and the bag is finally sealed and discarded into regular household trash. In use, the drugs will dissolve into the water and get adsorbed to the activated carbon in an irreversible manner and get deactivated^18^. In a previous study, it has been shown that such system has been able to efficiently deactivate some psychoactive medications^19^. This study’s aim was to evaluate the efficiency of this drug disposal system for deactivating other medications including alprazolam tablets, temazepam capsules, and zolpidem tablets (Fig. 1).

## Materials and Methods

### Chemicals

Analytical standards for alprazolam and temazepam were obtained from Sigma-Aldrich (St. Louis, MO, USA). Zolpidem tartrate standard was purchased from 2 A PharmaChem (Lisle, IL, USA). Methanol (MeOH) and acetonitrile (ACN) were of HPLC grade and supplied by Pharmco-Aaper (Shelbyville, KY, USA). Deionized (DI) water (Milli-Q^®^ Direct 8/16 System) was used. Alprazolam 2 mg tablets were obtained from Sandoz Inc. (Princeton, NJ, USA) and Sun Pharmaceutical Industries (Mumbai, India). Zolpidem tartrate 5 mg tablets were provided by TEVA Pharmaceuticals (Sellersville, PA, USA). Temazepam 30 mg capsules were purchased from Sandoz Inc. (Princeton, NJ, USA). Deterra^®^ Drug Deactivation System was provided by Verde Technologies (Minnetonka, MN, USA).

### HPLC analysis

#### Instrumentation and analytical conditions

The chromatographic analysis was carried out on a Waters (Milford, MA, USA) Alliance e2795 Separations Module HPLC, equipped with a quaternary pump, an autoinjector and a column heater coupled with a photodiode array detector (Waters 2998). Data acquisition and processing were performed using Empower 3 software. The methods utilized an isocratic reverse phase elution at ambient temperature. The analytical conditions for the three analytes are shown in Table 1. All mobile phases were freshly prepared, filtered through nylon membrane with a pore size of 0.22 µm (Millipore, USA), and sonicated (Fisher Scientific FS60H, Pittsburgh, PA) for 30 minutes before use to degas entrapped air bubbles.

**Table 1:** The optimized chromatographic conditions for analysis of Alprazolam, Temazepam, and Zolpidem.

Drug	Stationary Phase	Mobile Phase	Flow rate (mL/min)	Run time (min)	Injection Volume (µL)	Detection wavelength (nm)	Retention Time (min)
Alprazolam	Phenomenex Kinetex C18 (4.6 × 250 mm 5 µm)	ACN: KH_2_PO_4_, 10 mM pH = 4.5 (40:60 v/v)	1.0	10	20	221	4.7
Temazepam	Phenomenex Kinetex C18 (4.6 × 250 mm 5 µm)	ACN: KH_2_PO_4_, 20 mM pH = 3.0 (45:55 v/v)	1.0	10	20	230	4.8
Zolpidem	Agilent Eclipse Plus C18 (4.6 × 150 mm 5 µm)	ACN: KH_2_PO_4_, 25 mM pH = 6.0 (40:60 v/v)	1.0	10	20	243	5.1

ACN: Acetonitrile.

#### Preparation of stock and standard solutions

Primary stock solutions (1 mg/mL) of all three drugs were prepared in fresh DI water. These stock solutions were further diluted with DI water to prepare a series of working standards in the concentration range of 0.1–100 µg/mL and were used as calibration standards.

#### Linearity

The methods were validated in terms of the analytical parameters of linearity, specificity, precision, accuracy, limit of detection (LOD), and limit of quantification (LOQ) according to the International Conference on Harmonization (ICH) guideline on validation of analytical procedures^20^. Ten-point calibration curves (n = 3) were constructed in the concentration range of 0.1–100 µg/mL. Each standard solution was analyzed using the conditions described in Table 1.

#### Specificity

For all the analytes, the method’s specificity was assessed to ensure the analyte’s peak is not affected by the presence of other excipients and chemicals.

#### Precision and accuracy

Precision and accuracy were determined using quality control samples at three pre-defined concentration levels (low, medium, and high) within the range of linearity. Repeatability (intra-day precision) was reported as the coefficient of variation (%CV) of responses for six replicate injections of each concentration. Intermediate precision (inter-day) was measured by comparing the responses of each concentration on three different days and the results were reported as %CV. Accuracy from all determinations for three concentration levels was reported as the percent of analyte determined by an assay from a known injected concentration.

#### Limits of detection (LOD) and quantitation (LOQ)

The LOD is defined as the lowest concentration of the analyte which can be differentiated from background noise by the analytical method. The LOQ is defined as the lowest concentration which can be measured and quantified by the method with acceptable accuracy, precision. The LOD and LOQ were calculated by the following formulas:

$$\begin{array}{c}{\rm{L}}{\rm{O}}{\rm{D}}=3.3\,{\rm{\sigma }}/{\rm{S}}\\ {\rm{L}}{\rm{O}}{\rm{Q}}=10.0\,{\rm{\sigma }}/{\rm{S}}\end{array}$$

where σ is the standard deviation of intercept values acquired from drawn calibration curves (n = 3) and S is the slope.

### Deactivation study

The deactivation of the pharmaceutical dosage forms was studied on three model CNS depressant medications including alprazolam 2 mg tablet, temazepam 30 mg capsule, and zolpidem tartrate 5 mg tablet. Medications (ten alprazolam tablets, ten temazepam capsules, ten zolpidem tablets) were placed in separate Deterra^®^ drug deactivation pouches. Deterra^®^ system is a re-sealable plastic pouch (15 cm × 10 cm), containing 15 grams of granulated activated carbon enclosed by a bag of a water-soluble film. To each pouch, 50 mL of warm (43 °C) tap water was added. For the proper mixture, pouches were tilted back and forth ten times. Followed by a 30 second waiting period to allow the air bubbles to get released from carbon, pouches were sealed and then stored in an upright and undisturbed position at room temperature. For each time point, two separate pouches were allocated, and samples were taken from the pouches (n = 2) at the following intervals: 8 h, 1, 2, 4, 7, 14, 21, and 28 days. At the time of sampling, pouches were shaken to ensure homogenous mixing and then were opened to take samples (1 mL). Collected samples were centrifuged at 12100 × *g* for 3 minutes, filtered through 0.45 µm nylon syringe filters, and finally analyzed using validated HPLC-UV methods to determine drug content. The ratio of deactivation was calculated using the following equation:

$$ \% \,{\rm{of}}\,{\rm{drug}}\,{\rm{deactivated}}=\frac{{\rm{Initial}}\,{\rm{drug}}\,{\rm{amount}}-{\rm{remaining}}\,{\rm{drug}}\,{\rm{amount}}\,{\rm{after}}\,{\rm{deactivation}}}{{\rm{Initial}}\,{\rm{drug}}\,{\rm{amount}}}\times 100$$

### Desorption study

Followed by the deactivation study, desorption or washout study was performed to investigate the potential and the extent to which the drug leaches out of the activated carbon.

After 28 days, the entire content of each pouch was transferred into an individual plastic bottle, followed by the addition of 200 mL of deionized water. The containers were shaken for one hour at 150 rpm and then stored at room temperature for 23 hours. At this point, samples were collected from water washouts, they were then filtered and analyzed by HPLC-UV. In the next step, water content was replaced with 250 mL of 30% ethanol, shook for one hour, and stored for 23 hours at room temperature. Samples from ethanol washouts were filtered and then analyzed by HPLC-UV. The ratio of desorption was calculated using the following equation:

$$ \% \,{\rm{of}}\,{\rm{drug}}\,{\rm{desorbed}}=\,\frac{\text{Drug}\,\text{amount}\,{\rm{in}}\,{\rm{wash}}\,{\rm{out}}\,{\rm{solution}}}{{\rm{Initial}}\,{\rm{drug}}\,{\rm{amount}}}\times 100$$

## Results and Discussion

### HPLC method

**Figure 2:**
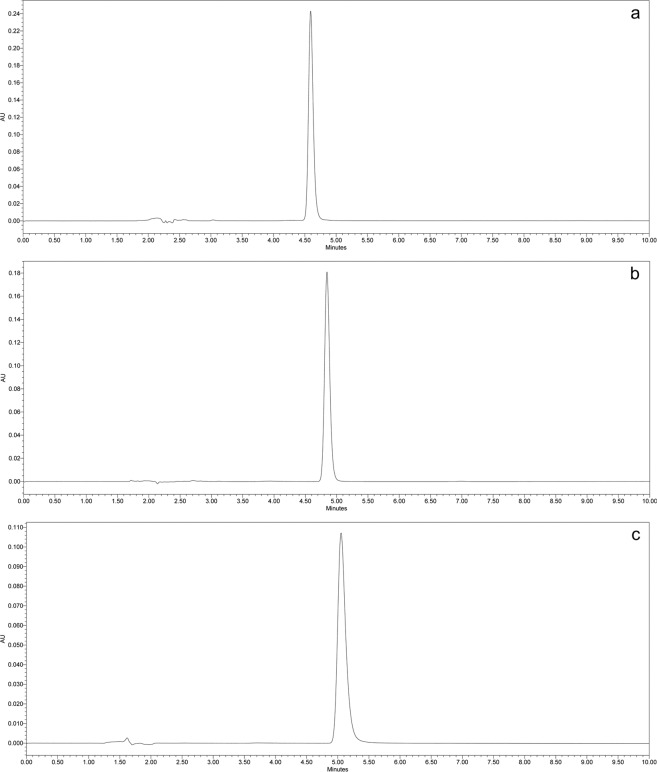
Representative chromatograms of (**a**) Alprazolam, (**b**) Temazepam, (**c**) Zolpidem at a concentration of 10 µg/mL.

A simple and rapid RP-HPLC method was successfully developed and validated for each drug. Chromatographic conditions were optimized to obtain a sharp symmetrical peak with reasonable retention time. The entire run time was short (10 min) for all three analytes and the retention times for alprazolam, temazepam, zolpidem was 4.7, 4.8 and 5.1 min, respectively. Specificity of analytical methods for each analyte was established, and no interfering peak from excipients or other compounds was observed at or adjacent to the retention time for all the analytes. Representative chromatograms for each drug is shown in Fig. 2.

A ten-point calibration curve was obtained using calibration standards for each analyte. All calibration curves were found linear (R^2^ ≥ 0.999) in the concentration range of 0.1–100 µg/mL. The slopes and intercepts were calculated using the plot of peak area versus drug concentration. The calculated LOQ and LOD concentrations established that the methods had sufficient sensitivity (Table 2).

**Table 2:** Validation parameters (linearity, LOD, LOQ) of HPLC methods for alprazolam, temazepam, and zolpidem.

Validation parameters	Alprazolam	Temazepam	Zolpidem
Range (µg/mL)	0.1–100	0.1–100	0.1–100
Regression equation	y = 127913x + 18807	y = 116570x − 22701	y = 101849x − 34244
Correlation coefficient (R^2^)	R² = 0.9999	R² = 0.9996	R² = 0.9996
σ of intercepts	6520.91	16411.10	2939.95
Average of slopes	135658.67	112817.33	101784.67
Calculated LOD (µg/mL)	0.16	0.48	0.10
Calculated LOQ (µg/mL)	0.48	1.45	0.29

LOD: Limit of Detection, LOQ: Limit of Quantification.

Accuracy and precision were determined by six injections of three levels of concentration for intra-day and total injection of twelve on three separate days for the inter-day validation. For all the analytes, the intra- and inter-day precision (%CV) at three concentration levels were observed to be less than 10%. Accuracy values for all the drugs were within the range of 92–103% (Table 3). All the validation parameters of the three analytes were found to be within the specified limits. The developed HPLC methods were specific, sensitive, accurate, precise, and reproducible. Hence, the methods were suitably employed o assay the analyte amount in deactivation and desorption studies.

**Table 3:** Validation parameters (inter- and intra-day precision and accuracy) of HPLC methods for alprazolam, temazepam, and zolpidem.

Analyte	Expected Conc. (µg/mL)	Intra-day (n = 6)			Inter-day (n = 12)		
		Measured Conc. (µg/mL, Mean ± SD)	Accuracy (%, Mean ± SD)	%CV	Measured Conc. (µg/mL, Mean ± SD)	Accuracy (%, Mean ± SD)	%CV
Alprazolam	10	10.04 ± 0.08	100.40 ± 0.80	0.80	10.05 ± 0.09	100.48 ± 0.92	0.92
	25	25.82 ± 0.48	103.28 ± 1.93	1.87	25.65 ± 0.60	102.61 ± 2.39	2.33
	50	51.10 ± 0.16	102.20 ± 0.33	0.32	50.67 ± 0.49	101.34 ± 0.97	0.96
Temazepam	10	9.92 ± 0.05	99.22 ± 0.55	0.55	9.23 ± 1.01	94.70 ± 5.87	6.20
	25	25.25 ± 0.24	100.99 ± 0.95	0.94	24.25 ± 1.36	97.00 ± 5.44	5.61
	50	47.91 ± 0.52	95.81 ± 1.04	1.09	49.68 ± 2.57	99.35 ± 5.14	5.17
Zolpidem	10	10.07 ± 0.06	100.73 ± 0.60	0.60	10.08 ± 0.07	100.78 ± 0.67	0.66
	25	23.11 ± 0.09	92.43 ± 0.34	0.37	23.12 ± 0.18	92.46 ± 0.71	0.77
	50	50.00 ± 0.30	99.99 ± 0.59	0.59	49.98 ± 0.30	99.95 ± 0.60	0.60

### Deactivation study

**Figure 3:**
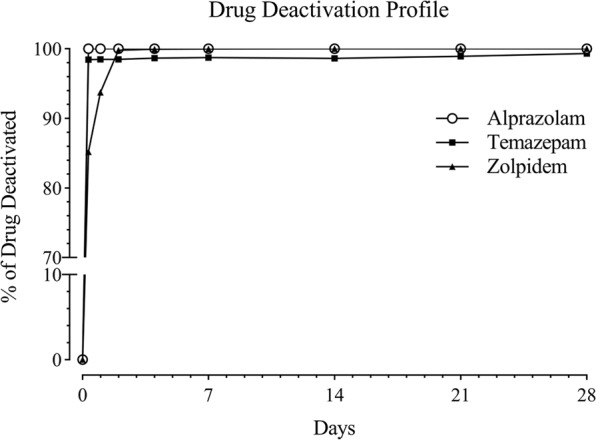
Deactivation profile of alprazolam, temazepam, and zolpidem over the course of 28 days.

Deactivation of the sedative model drugs (alprazolam, temazepam, zolpidem) using the activated carbon-based drug disposal system was investigated over the course of 28 days. Once the dosage forms are placed into the pouches and water is added, the drug is going to be released from the dosage form and gets absorbed to the highly porous structure of activated carbon and gets deactivated. The deactivation profile for all three drugs is shown in Fig. 3. After 8 hours, alprazolam, temazepam, and zolpidem were deactivated by the extent of 100.00, 98.46, and 85.17% respectively. All drugs continued to be deactivated over time, and by the end of study at the 28th day, an average of 99.77 ± 0.39% of the drug content was effectively deactivated by the drug disposal system. The residual amount of drug for temazepam was less than 1.00%, and no residual amount was found for alprazolam and zolpidem.

### Desorption study

This study was carried out to investigate the possible desorption of tested medications from activated carbon. Contents of each pouch went through washing procedures with large volumes of aqueous and organic solvents to simulate landfill situations. Results showed that in all washout studies less than 0.5% of the drug leached out which proves that this disposal system can adsorb the drugs in an irreversible manner (Table 4). Once the drug molecules are bond to the surface of active carbon particles, the intermolecular forces are strong enough to prevent the release of the molecules from the carbon and render the drug molecules inactive^16^.

**Table 4:** Desorption study results for alprazolam, temazepam, and zolpidem.

Medications	% Leached in water	% Leached in 30% ethanol
Alprazolam	0.00	0.00
Temazepam	0.05	0.04
Zolpidem	0.00	0.21

Findings of this study are in accordance with previous research performed regarding the use of activated carbon to deactivate medications. Activated has been shown to exert better efficiency as a universal drug deactivation agent as compared to the other agents such as sodium percarbonate, sodium carbonate, and zeolite^16^. Activated carbon has also been successfully used for deactivation of opioid medications including morphine solution, methadone, hydromorphone, and meperidine tablets. Average deactivation of more than 99.00% was observed, and less than 1.3% drug content leached out in desorption studies^21^. In another study diazepam tablets, buprenorphine sublingual films, and lorazepam tablets were deactivated by the average of more than 99.00% and the leached out amount was less than 0.7% in wash-out studies^19^.

Sedatives including benzodiazepines and Z-drugs (e.g., zolpidem) are widely prescribed to treat anxiety and insomnia. However, patients may misuse or abuse sedatives for self-medication or recreational purposes leading to intoxication or withdrawal syndromes, which may be fatal in either case^22^. Accumulation of unused or unexpired medications in households or improper disposal can lead to intentional misuse and abuse, accidental exposure and contaminating the environment. Results of this study and others as well indicate that an activated carbon-based drug deactivation system is capable of successful and irreversible deactivation of various medications and dosage forms. making it an efficient system in that users can easily discard of their unused medications by simply placing them in the pouch containing activated carbon, adding water, sealing the bag and discarding it to normal household trash.

## Concluding Remarks

The efficiency of an activated carbon-based drug disposal system in deactivating model sedative medications was evaluated. The deactivation system efficiently adsorbed and deactivated approximately 94% of the tested medications within 8 hours and over 99% by 28 days. Drug substances did not get released from activated carbon when washed out with large volumes of water or 30% ethanol indicating minimal environmental effect. Therefore, this unique system provides a simple, safe and an efficient drug disposal system which can be used in households and healthcare settings to deactivate unused or expired medications.

## Data Availability

The datasets generated during and/or analysed during the current study are available from the corresponding author on reasonable request.
